# Genome sequence, antibiotic resistance genes, and plasmids in a monophasic variant of *Salmonella typhimurium* isolated from retail pork

**DOI:** 10.1128/mra.00754-23

**Published:** 2024-05-03

**Authors:** Anahita Ghorbani Tajani, Aniket Sharma, Nicolas Blouin, Bledar Bisha

**Affiliations:** 1 Department of Animal Science, University of Wyoming, Laramie, Wyoming, USA; 2 INBRE Data Science Core, University of Wyoming, Laramie, Wyoming, USA; University of Maryland School of Medicine, Baltimore, Maryland, USA

**Keywords:** genome sequence, antibiotic resistance genes, plasmids, *Salmonella typhimurium*, foodborne pathogens

## Abstract

A *Salmonella* isolate from retail pork was whole genome sequenced using Illumina NovaSeq6000, with a 5,320,119 bp genome and 51.06% GC content. Several antibiotic resistance genes and plasmids, including *bla*_TEM-1_, *aac(6')-IIc*, IncHI2, and p0111 were obtained from subsequent analysis. These findings provide vital insights into generic determinants of antimicrobial resistance (AMR) in this foodborne pathogen.

## ANNOUNCEMENT

Antimicrobial resistance (AMR) is a pressing public health threat that demands attention and understanding of antibiotic resistance genes (ARGs) in bacterial pathogens (1). This announcement reports the genome sequence, detection, and characterization of ARGs and plasmids in a *Salmonella* isolate from retail pork in Laramie, Wyoming. The cultural isolation and comprehensive molecular analysis were performed following the National Antimicrobial Resistance Monitoring System protocol (2), which is a standard protocol for monitoring AMR in foodborne pathogens.

The *Salmonella* isolates from pork samples were subjected to isolation involving the enrichment of one pork chop from each package with 250 mL buffered peptone water. After mechanical shaking and overnight incubation at 35°C, the samples were transferred to Rappaport-Vassiliadis R10 (RVR10, Remel) broth medium and incubated at 42°C for 20–24 hours. Subsequently, the enriched cultures were streaked onto XLT-4 plates (remel) and incubated at 35°C (2). Isolate confirmation as *Salmonella enterica* was performed via matrix-assisted laser desorption/ionization-time of flight (MALDI-TOF-MS) in the form of a Bruker Microflex LRF instrument Biotyper-version 3, and the serovar determination was performed with SeqSero-1.2. Following confirmation, DNA extraction and purification were carried out using the DNAeasy PowerLyzer Microbial Kit (Qiagen) following the manufacturer’s protocols. Single colonies were subsequently subcultured in 10 mL of BHI and incubated at 37°C overnight. DNA quality and yield were assessed with a Qubit 2.0 Fluorometer (Thermo Scientific, Waltham, MA).

High-throughput sequencing was conducted at CD Genomics (New York) using an Illumina NovaSeq6000 (150 bp paired-end reads, 500 ng genomic DNA/sample). Library preparation used the NEBNext Ultra DNA Library Prep Kit for Illumina (NEB, USA) following standard protocols. DNA samples were sonicated to 350 bp, end-polished, A-tailed, ligated with adaptors, and PCR amplified. Library size was analyzed using Agilent 2100 Bioanalyzer and quantified via real-time PCR.

Sequencing achieved a coverage of 100× per sample. For quality control, FastQC-version 0.12.0 (3) was used, and contigs assembly was performed using SPAdes-version 3.15.5 (4). Genome annotation utilized Prokka-version 1.14.5 (5). For assembly statistics, including the number of contigs and N_50_ values, were determined from SPAdes assembler output. ARGs were identified by ABRicate-version 0.2-0 (6) utilized, plasmid presence was assessed using PlasmidFinder-version 2.0.1 (7), using all the default parameters unless specified.

*Salmonella* isolates exhibited several ARG’s and plasmids including IncHI2 (Identity: 100%, Query/Template length: 327/327, Contig: NZ_JARFRZ010000037.1), IncHI2A (Identity: 100%, Query/Template length: 630/630, Contig: NZ_JARFRZ010000032.1), and p0111 (Identity: 98.08%, Query/Template length: 885/885, Contig: NZ_JARFRZ010000014.1). Ten detected ARG’s include *bla*_TEM-1_, *aac(6')-IIc*, *aac(3)-II*, *arr-269927220*, *ere(A*), *bla*_SHV-12_, *tet(D*), *sul1*, *qnrB2*, and *aadA2*.

Genomic characterization revealed a total genome size of 5,320,119 bp with a GC content of 51.06%. The draft genome consisted of 87 contigs, with a contig N_50_ of 223,843 bps and a contig L_50_ of 9. The genome also comprised 79 scaffolds, with scaffold N_50_ of 243,627 bps and a scaffold L_50_ of 7. Furthermore, sequencing produced 2,432,783 bps reads.

## Data Availability

The study’s genome assembly is deposited in the GenBank database (https://www.ncbi.nlm.nih.gov/genbank/) under the accession number JARFRZ000000000.1. Raw sequencing data are available in the Sequence Read Archive (SRA) under project number PRJNA939729 with accession number SRR25618345. Additionally, detailed annotation information is available at https://doi.org/10.6084/m9.figshare.25052813.v1. Access to the genome assembly and its associated data can be acquired using the provided accession numbers.
